# Magnetic anisotropy and high-frequency property of flexible FeCoTa films obliquely deposited on a wrinkled topography

**DOI:** 10.1038/s41598-017-03288-6

**Published:** 2017-06-06

**Authors:** Jincai Li, Qingfeng Zhan, Shuanglan Zhang, Jinwu Wei, Jianbo Wang, Minjie Pan, Yali Xie, Huali Yang, Zheng Zhou, Shuhong Xie, Baomin Wang, Run-Wei Li

**Affiliations:** 1 CAS Key Laboratory of Magnetic Materials and Devices and Zhejiang Province Key Laboratory of Magnetic Materials and Application Technology, Ningbo Institute of Materials Technology and Engineering, Chinese Academy of Sciences, Ningbo, 315201 People’s Republic of China; 2 Key Laboratory of Low Dimensional Materials and Application Technology of Ministry of Education, School of Materials Science and Engineering, Xiangtan University, Xiangtan, Hunan 411105 People’s Republic of China; 3 Key Laboratory for Magnetism and Magnetic Materials of the Ministry of Education, Lanzhou University, Lanzhou, 730000 People’s Republic of China

## Abstract

We investigated the magnetic anisotropy and the high-frequency property of flexible Fe_60_Co_26_Ta_14_ (FeCoTa) thin films obtained by oblique sputtering onto a wrinkled surface. The sinuously wrinkled topography is produced by growing Ta layer on a pre-strained polydimethylsiloxane (PDMS) membrane. Due to the enhanced effect of shadowing, the oblique deposition of FeCoTa layer gives rise to a shift of wrinkle peak towards the incident atomic flux. With increasing the PDMS pre-strain or increasing the oblique sputtering angle, both the uniaxial magnetic anisotropy and the ferromagnetic resonance frequency of FeCoTa films are enhanced, but the initial permeability decreases. The magnetization reversal mechanism of wrinkled FeCoTa films can be interpreted by a two-phase model composed of both coherent rotation and domain wall nucleation. With the enhancement of uniaxial magnetic anisotropy, the domain wall nucleation becomes pronounced in FeCoTa films.

## Introduction

Nowadays, magnetic thin films have been widely used in various kinds of high-frequency devices such as thin-film inductors, microwave filters, and microwave absorbers, etc^1, 2^. In order to ensure an operating frequency in the gigahertz range for microwave devices, the ferromagnetic resonance frequency of magnetic thin films should be well tuned to a very high value. It is well known that the ferromagnetic resonance frequency *f*_*r*_ of magnetic films is determined by the magnetic anisotropy *H*_*k*_ according to the Kittel’s equation ${f}_{r}=\frac{\gamma }{2\pi }\sqrt{{H}_{k}({H}_{k}+4\pi {M}_{s})}$, where *γ* is gyromagnetic ratio and *M*_*s*_ is saturation magnetization^3^. Conventionally, there are several methods to obtain an enhanced in-plane uniaxial magnetic anisotropy in magnetic films, such as oblique deposition^4–6^, magnetic field annealing^7–11^, and exchange bias^12–16^. Among them, the oblique deposition can be easily handled, thus has been widely employed in practice to promote the ferromagnetic resonance frequency of magnetic films^2, 17^. Because of a self-shadowing effect, oblique deposition of a magnetic film onto a flat and rigid substrate may result in the formation of grains in the film which are elongated perpendicular to the incident flux direction. Consequently, a uniaxial magnetic anisotropy with the easy axis perpendicular to the incident flux direction is induced during the growth of magnetic films^18^.

Recently, a lot of works have tried to produce a uniaxial magnetic anisotropy by modifying the surface morphology of magnetic thin films. For instance, Ki *et al*. reported that NiFe thin films with a triangular wave-like morphology grown on m-plane Al_2_O_3_ substrate display a remarkable uniaxial magnetic anisotropy^19^. Briones *et al*. produced a rippled Co film by the deposition on a wrinkled polydimethylsiloxane (PDMS) substrate, resulting in a uniaxial magnetic anisotropy oriented parallel to the wrinkles^20^. In the similar way, Zhang *et al*. reported an enhanced uniaxial magnetic anisotropy in FeGa films deposited on a wrinkled surface which is obtained by growing metallic layer on pre-stretched PDMS membrane^21^. As compared to the deposition on a flat substrate, the shadowing effect of oblique deposition onto a wrinkled surface can be significantly enhanced due to the sinuously oscillated topography, thus displaying a rather stronger magnetic anisotropy.

FeCo thin films have been widely used as soft magnetic materials in static and low frequency applications because of the high saturation magnetization^22^. For high frequency application, non-magnetic elements, such as Ta, Zr, Hf, are usually doped in FeCo alloys to effectively reduce the grain size of magnetic films and easily induce an in-plane uniaxial anisotropy in the films^23–25^. Among these FeCo-based alloys, FeCoTa films display excellent high-frequency behaviors with a resonance frequency beyond 5.1 GHz at room temperature, which is higher than that of FeCoZr, FeCoHf, and FeCoLu high-frequency magnetic films^26–28^. In this work, we select Fe_60_Co_26_Ta_14_ (FeCoTa) alloy to investigate the effect of oblique deposition on the surface morphology and magnetic property for magnetic films grown onto a wrinkled surface. Because of the enhanced shadowing effect by the wrinkled topography, the oblique deposition gives rise to a shift of wrinkle peaks and the FeCoTa thin films display a significantly uniaxial magnetic anisotropy. Both the enhancement of PDMS pre-strain and the increase of deposition angle could improve the uniaxial magnetic anisotropy and the ferromagnetic resonance frequency of FeCoTa films.

## Results

**Figure 1:**
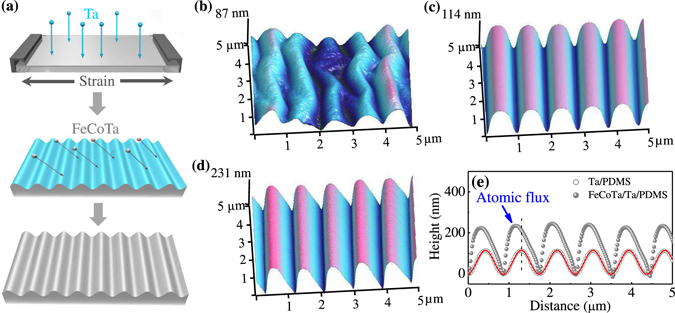
(**a**) Schematic plots for the steps of fabricating wrinkled magnetic FeCoTa films. (**b**) AFM image for a 150 nm FeCoTa film obliquely deposited at *φ* = 45° on a non-strained Ta(5 nm)/PDMS. (**c**) AFM image for a 5 nm Ta layer grown on a pre-strained 20% PDMS. (d) AFM image for a 150 nm FeCoTa film obliquely deposited at *φ* = 45° on the wrinkled topography of (**c**). (**e**) The cross-sectional views extracted from both Ta/PDMS (gray circle) in (**c**) and FeCoTa/Ta/PDMS (gray sphere) in (**d**). The red line is the sinusoidal fitting for the cross-sectional view of Ta/PDMS.

Figure 1(a) schematically shows the steps of fabricating wrinkled magnetic films. Before deposition, PDMS membranes were pre-stretched by a tensile pre-strain *ε*_pre_ up to 40%. A 5 nm Ta layer was grown as a buffer layer onto the pre-strained PDMS. After the PDMS pre-strain was released, a wrinkled topography appears on the surface of Ta/PDMS due to the mismatch of Young’s modulus between the PDMS substrate and the metallic layer^29^. Subsequently, a 150 nm FeCoTa film was obliquely deposited onto the wrinkled Ta/PDMS surface at an angle *φ* varying from 0° to 45° with respect to the surface normal and with the azimuth perpendicular to the wrinkles. A 3 nm Ta layer was additionally deposited on the samples as a protection layer to prevent oxidation. Figure 1(b) shows the surface morphology for a 5 nm Ta buffer layer grown on a non-strained PDMS, i.e., *ε*_pre_ = 0%, and then a 150 nm FeCoTa layer obliquely deposited at *φ* = 45°. A rather regular wrinkled topography with a wavelength of 1.01 μm and an amplitude of 87 nm is observed to be perpendicular to the direction of incident atomic flux. In contrast, we previously non-obliquely deposited FeGa layer on a freestanding PDMS. The film displays irregular wrinkles on the surface due to the random distribution of internal stress. Obviously, the formation of regular wrinkled topography for metallic films grown on a non-strained PDMS is because of the oblique deposition of FeCoTa layer. It is well known that due to a self-shadowing effect, the oblique incidence deposition results in a topography with grains elongated perpendicular to the incident flux direction. When FeCoTa atoms are obliquely deposited on a freestanding PDMS, the elongated grain structure produces a regularly distributed internal stress, which gives rise to the parallel aligned wrinkles perpendicular to the oblique incidence deposition.

When a 5 nm Ta buffer layer is grown onto a pre-stretched PDMS, after relaxing the pre-strain, the Ta/PDMS sample displays a much regular wrinkled topography. Figure 1(c) displays a typical wrinkled topography with a wavelength of 880 nm and an amplitude of 114 nm for Ta/PDMS sample grown with *ε*_pre_ = 20%. The cross-sectional view of the wrinkled topography can be well fitted to a sinusoidal curve, as shows in Fig. 1(e). The wavelength *λ* and the amplitude *A* of wrinkled Ta/PDMS topography can be described by an elastic model^29–32^ as $\lambda =\frac{1}{(1+{\varepsilon }_{pre}){(1+\xi )}^{\frac{1}{3}}}\cdot \frac{\pi {t}_{f}}{\sqrt{{\varepsilon }_{c}}}\,{\rm{and}}\,A=\frac{{t}_{f}}{\sqrt{1+{\varepsilon }_{pre}}{(1+\xi )}^{\frac{1}{3}}}\cdot \sqrt{\frac{{\varepsilon }_{pre}}{{\varepsilon }_{c}}-1}$, where ${\varepsilon }_{c}=0.52{[\frac{{E}_{s}(1-{\nu }_{f}^{2})}{{E}_{f}(1-{\nu }_{s}^{2})}]}^{\frac{2}{3}}$ is a certain threshold strain for buckling and has to be exceeded for obtaining a wrinkle pattern, $\xi =\frac{5}{32}{\varepsilon }_{pre}(1+{\varepsilon }_{pre})$, *t*_ƒ_ is the total thickness of metal layers. *E*_*s*_ and *ν*_*s*_ are the Young’s modulus and Poisson’s ratio for PDMS membranes and the same as the *E*_*ƒ*_, *ν*_*ƒ*_ for Ta films, respectively. Based on the previously reported elastic parameters for Ta bulks (*E*_*ƒ*_ = 100 GPa, *ν*_*ƒ*_ = 0.3) and PDMS membranes (*E*_*s*_ = 1 MPa, *ν*_*s*_ = 0.5), we may predict the wavelength and the amplitude of the wrinkled Ta/PDMS surface. For example, a wavelength of 779 nm and an amplitude of 121 nm are estimated for the topography of Ta(5 nm)/PDMS grown with *ε*_pre_ = 20%, which agree well with the experimentally obtained results^21^. Since the elastic parameters almost cannot be changed, in order to obtain a wrinkled surface with a desired wavelength and amplitude, both the PDMS pre-strain and the thickness of metal layer need to be well controlled.

After obliquely depositing a 150 nm FeCoTa layer onto the wrinkled Ta/PDMS surface, the wavelength approximately keeps unchanged, but the amplitude is increased. Figure 1(d) typically indicates the surface topography for a 150 nm FeCoTa film obliquely deposited at *φ* = 45° on a wrinkled Ta/PDMS surface obtained by using *ε*_pre_ = 20%. As shown in Fig. 1(e), the corresponding cross-sectional view is no longer fluctuated according to a sine function. The peaks of wrinkles are shifted by 127 nm toward the incident atomic flux. The magnetic layer on the wrinkle side faced to the oblique sputtering become much thicker than that on the opposite side due to the enhanced shadowing effect by the wrinkled morphology. Although we use a fixed incidence angle to obliquely deposit FeCoTa layer, the sinuously oscillated morphology of Ta/PDMS makes the atomic flux displaying different incidence angles, which consequently gives rise to the inhomogeneous thickness and changes the sinuously wrinkled surface. For a fixed *ε*_pre_, the decrease of the oblique sputtering angle may reduce the shift of wrinkles. For fixing *φ* = 45° to grow FeCoTa but change the pre-strain of PDMS to obtain different wrinkled Ta/PDMS surfaces, the wavelength of wrinkles determined by the Ta buffer layer decreases from 929 to 655 nm with increasing *ε*_pre_ from 5% to 40%, but the corresponding amplitude increases from 151 to 289 nm and the shift of peak gradually decreases from 147 to 19 nm. Obviously, the increase of either the oblique deposition angle or the pre-strain applied on PDMS may enhance the shadowing effect, leading to an inhomogeneous thickness and a non-sinuously wrinkled topography.

**Figure 2:**
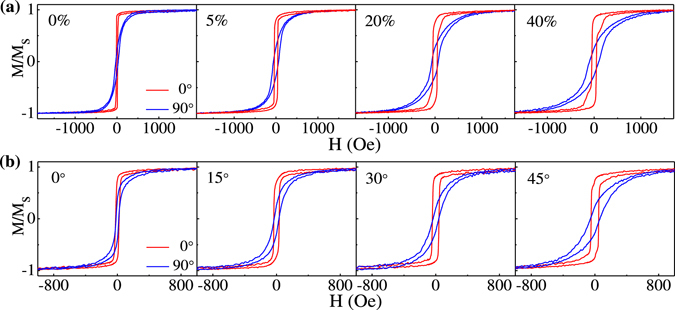
Hysteresis loops with magnetic field applied parallel (*θ* = 0°) and perpendicular (*θ* = 90°) to wrinkles for FeCoTa films grown (**a**) with different pre-strains of 0%, 5%, 20%, 40% and (**b**) with different deposition angles of 0°, 15°, 30°, 45°.

Figure 2(a) shows the hysteresis loops measured with an in-plane magnetic field applied parallel (*θ* = 0°) and perpendicular (*θ* = 90°) to the wrinkles for FeCoTa films grown with different *ε*_pre_ and a fixed *φ* = 45°. All the wrinkled FeCoTa films display a uniaxial magnetic anisotropy with the easy axis along the wrinkles. When *ε*_pre_ is varied from 0% to 40%, the coercivity measured along the easy axis increases from 15 to 49 Oe. By means of calculating the difference of the area enclosed between the hysteresis loops measured along the easy and hard axes^33^, the magnetic anisotropy is estimated to increase from 3.68 × 10^4^ to 1.30 × 10^5^ erg/cm^3^. Figure 2(b) shows the hysteresis loops for FeCoTa films grown with different *φ* and a fixed *ε*_pre _ = 30%. With increasing *φ* from 0° to 45°, the coercivity measured along the easy axis correspondingly increases from 24 to 47 Oe and the uniaxial magnetic anisotropy increases from 2.16 × 10^4^ to 1.21 × 10^5^ erg/cm^3^. It is well known that the method of oblique deposition may result in a uniaxial magnetic anisotropy with an easy axis perpendicular to the incident atomic flux. The oblique deposition onto the sinuously wrinkled surface may enhance the effect of shadowing and significantly increase the uniaxial magnetic anisotropy of magnetic films. On the other hand, the wavy morphology of wrinkled magnetic films can produce an additional surface magnetic anisotropy. When a saturation magnetic field is applied perpendicular to the wrinkles, FeCoTa moments are aligned parallel to the film plane, which creates magnetic charges on the film surface. The dipolar interaction between the magnetic charges acts as a coupling field favoring parallel alignment of magnetization, inducing a surface anisotropy with easy axis along the wrinkles. The strength of surface anisotropy is estimated less than 3 × 10^3^ erg/cm^3^, which is by far less than the contribution from the oblique deposition^21, 34, 35^.

**Figure 3:**
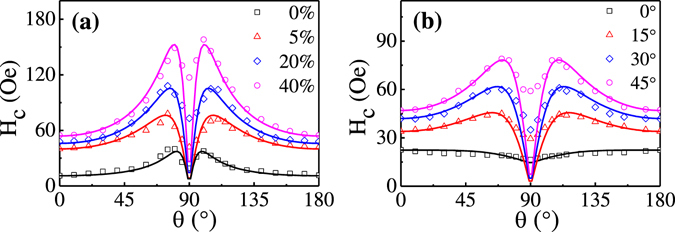
Coercivity *H*_*c*_ as a function of magnetic field orientation *θ* and the corresponding fitting lines by a two-phase model for FeCoTa films grown (**a**) with different pre-strains of 0%, 5%, 20%, 40% and (**b**) with different deposition angles of 0°, 15°, 30°, 45°.

Figure 3(a) and (b) show the angular dependence of coercivity for FeCoTa films grown with different *ε*_pre_ and with different *φ*, respectively. Both of them exhibit a uniaxial symmetry about the directions parallel (*θ* = 0°) and perpendicular (*θ* = 90°) to the wrinkles, confirming the uniaxial magnetic anisotropy in the wrinkled FeCoTa films. For the magnetic field orientation *θ* rotated away from the easy axis (*θ* = 0°), the coercivity first increases but decreases sharply when approaching the hard axis (*θ* = 90°). This kind of angular dependent behaviors cannot be solely interpreted by either the coherent rotation or the domain wall nucleation. Conventionally, the Stoner-Wohlfarth model considering the coherent rotation of magnetization predicts a monotonous decrease of coercivity with increasing *θ* from 0° to 90° ^36, 37^. In contrast, the coercivity predicted by the Kondorsky model based on the domain wall nucleation and propagation monotonously increases with increasing *θ* from 0° to 90° and diverges for *θ* close to 90° ^38^. A two-phase model composed of both coherent rotation and domain wall nucleation is usually employed to account for the magnetization reversal in polycrystalline magnetic films^39–42^. In this model, the application of magnetic field closed to the easy axis results in the nucleation of domain walls. For magnetic field applied close to the hard axis, the magnetization coherently rotates. The coercivity in a two-phase system can be described as^40, 42^

1 $${H}_{C}(\theta )={H}_{C}(0^\circ )\frac{({N}_{x}+{N}_{N})\cos \,\theta }{{N}_{z}{\sin }^{2}\,\theta +({N}_{x}+{N}_{N}){\cos }^{2}\,\theta },$$

where *N*_z_ and *N*_x_ are the demagnetizing factors in the directions parallel (*θ* = 0°) and perpendicular (*θ* = 90°) to the wrinkles, respectively. *N*_N_ = *H*_k_/*M*_s_ is an effective demagnetizing factor. If the ratio *y* = (*N*_N_ + *N*_x_)/*N*_z_ is close to zero, the magnetization reversal mechanism in the two-phase system is dominated by the coherent rotation. For an infinite *y*, the magnetization reversal mechanism in the two-phase system is mediated by the domain wall nucleation. By using the two-phase model, the experimentally obtained angular dependence of coercivity can be well fitted, as shown in Fig. 3(a) and (b). The fitting parameter of *y* increases from 13 to 31 for the samples grown with increasing *ε*_pre_ from 0% to 40% and with a fix *φ* = 45°. For the samples grown with a fix *ε*_pre_ = 30%, *y* increase from 2 to 10 with increasing *φ* from 0° to 45°. The enhanced factor of *y* by increasing either the PDMS pre-strain or the oblique sputtering angle indicates that the enhancement of uniaxial magnetic anisotropy makes the domain wall nucleation but not the coherent rotation dominative in the wrinkled FeCoTa films. It should be noted that there is an obvious difference between the calculation value and the experimental value of *H*_*c*_ at *θ* = 90°. It is because the two-phase model considers a single-crystal system with an ideal uniaxial anisotropy and predicts *H*_*c*_ = 0 at *θ* = 90°. However, for polycrystalline films, the distribution of grain easy axis leads to *H*_*c*_ ≠ 0 at *θ* = 90° ^40, 42^. Additionally, for our polycrystalline FeCoTa films, the magnetic moments on the wrinkled surface morphology are not strictly parallel to the external magnetic field applied perpendicular to the wrinkles (*θ* = 90°), but make a small angle less than 10°, which also results in *H*_*c*_ ≠ 0 at *θ* = 90°.

**Figure 4:**
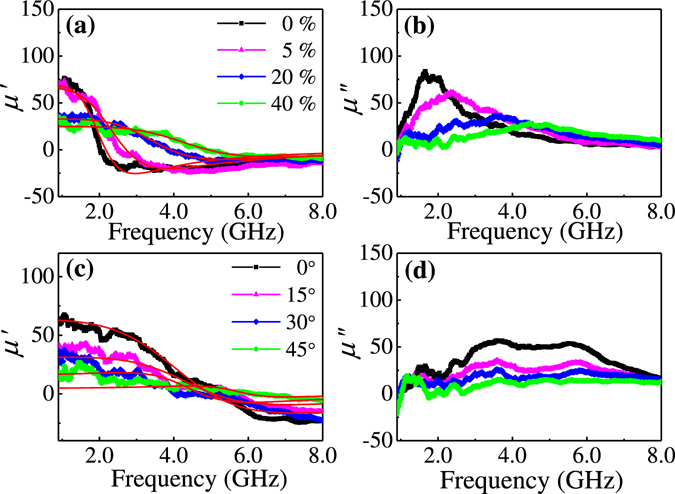
(**a**) Real and (**b**) imaginary permeability spectra obtained at zero bias field for FeCoTa films grown with different pre-strains. (**c**) Real and (**d**) imaginary permeability spectra obtained at zero bias field for FeCoTa films grown with different deposition angles. The red lines are the fitting by the LLG equation.

**Figure 5:**
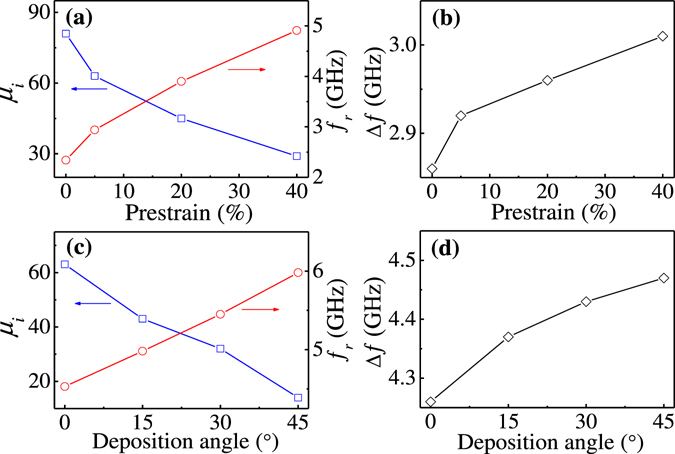
(**a**) Initial permeability, (**a**) ferromagnetic resonance frequency, and (**b**) frequency linewidth for FeCoTa films grown at *φ* = 45° and with different pre-strains. (**c**) Initial permeability, (**c**) ferromagnetic resonance frequency, and (**d**) frequency linewidth for FeCoTa films grown with *ε*_pre_ = 30% and with different deposition angles.

Figure 4 presents the dynamic real (*μ*′) and imaginary (*μ*″) permeability spectra at zero bias field for the wrinkled FeCoTa films measured in the frequency range from 0.9 to 8 GHz. The complex permeability can be described by solving the Landau-Lifshitz-Gilbert (LLG) equation as^43^

2 $$\mu ^{\prime} ={\rm{1}}+{x}_{0}\frac{1-(1+{\alpha }^{2}){(\frac{f}{{f}_{r}})}^{2}}{{[1-(1+{\alpha }^{2}){(\frac{f}{{f}_{r}})}^{2}]}^{2}+{[2\alpha (\frac{f}{{f}_{r}})]}^{2}},$$

3 $$\mu ^{\prime\prime} ={x}_{0}\frac{{[2\alpha (\frac{f}{{f}_{r}})]}^{2}}{{[1-(1+{\alpha }^{2}){(\frac{f}{{f}_{r}})}^{2}]}^{2}+{[2\alpha (\frac{f}{{f}_{r}})]}^{2}},$$

where *χ*_*0*_ = *μ*_i_ − 1 is the initial susceptibility, *α* is the damping parameter, and *ƒ* is the operation frequency. Taking α, *χ*_0_, and *f*_*r*_ as the fitting parameters, the experimentally obtained real and imaginary permeability spectra for the wrinkled FeCoTa films can be fitted. For the films grown at *φ* = 45°, with *ε*_pre_ increasing from 0% to 40%, *μ*_i_ decreases from 81 to 29 but *ƒ*_*r*_ increases from 2.34 to 4.91 GHz, as shown in Fig. 5(a). The opposite features between the *ε*_pre_ dependence of *μ*_i_ and ƒ_*r*_ can be explained by using the Snoek-Archer’s limit: $({\mu }_{i}-1)\,{f}_{r}^{2}={[(\gamma /2\pi )4\pi {M}_{s}]}^{2}$ ^44, 45^. For a magnetic thin film with a certain *M*_*s*_, the increase of ƒ_*r*_ may lead to the decrease of *μ*_i_. Using the relation^46^ ${\rm{\Delta }}f=\gamma \alpha (4\pi {M}_{s}+2{H}_{k})/2\pi$, the fitting value of *α* is obtained as 0.1, the frequency linewidth *Δƒ* increases from 2.86 to 3.01 GHz with increasing *ε*_pre_ from 0% to 40%, as shown in Fig. 5(b). For FeCoTa films grown at a fixed *ε*_pre_ = 30%, with increasing *φ* from 0° to 45°, *μ*_i_ decrease from 63 to 14 and *ƒ*_*r*_ increases from 4.53 to 5.98 GHz, as displayed in Fig. 5(c). The frequency linewidth *Δƒ* correspondingly increases from 4.26 to 4.47 GHz, as shown in Fig. 5(d). The fitting value of α is about 0.15 which indicates a large magnetic loss. Thus, the imaginary permeability spectra cannot be well fitted by the LLG formula.

### Summary

In summary, we reported a method to fabricate flexible magnetic films with a remarkable uniaxial magnetic anisotropy by combining the self-assembled wrinkled surface and the oblique deposition. The sinuously wrinkled topography is produced by growing a non-magnetic Ta layer on a pre-stretched PDMS membrane. Due to the sinuously wrinkled topography, the shadowing effect is enhanced during the oblique deposition of magnetic FeCoTa films, leading to a remarkable uniaxial magnetic anisotropy and an enhanced ferromagnetic resonance frequency. The magnetization reversal mechanism of wrinkled FeCoTa films can be interpreted by a two-phase model composed of both coherent rotation and domain wall nucleation.

## Methods

PDMS membranes with a thickness of 360 μm were pre-stretched by a tensile pre-strain *ε*_pre_ up to 40% using a home-made stretching apparatus. A 5 nm Ta layer was grown onto the pre-strained PDMS by using a DC magnetron sputtering system with a base pressure better than 5 × 10^−5^ Pa. Then the samples were taken out of the vacuum chamber and the PDMS pre-strain was subsequently released. A wrinkled topography appears on the surface of Ta/PDMS due to the mismatch of Young’s modulus between the PDMS substrate and the metallic layer. Subsequently, we again introduced the samples into the sputtering chamber and obliquely deposited a 150 nm FeCoTa film onto the wrinkled Ta/PDMS surface at different growth angles with respect to the surface normal and with the azimuth perpendicular to the wrinkles. Prior to be taken out of the vacuum chamber, a 3 nm Ta layer was non-obliquely deposited on the samples as a protection layer to prevent oxidation. The thicknesses of Ta and FeCoTa layers were controlled by the deposition time and were calibrated by X-ray reflectivity. The surface morphology was characterized by an atomic force microscope (AFM, Veeco Dimension 3100V). The hysteresis loops were measured by using a vibrating sample magnetometer (VSM, Lakeshore 7410). The permeability spectra were obtained at zero bias field by using a vector network analyzer (Agilent E8363B) with a shorted microstrip transmission-line perturbation method. All the measurements were conducted at room temperature.

## References

[1] Phuoc NN, Ong CK (2013). Anomalous temperature dependence of magnetic anisotropy in gradient-composition sputterred thin films. Adv. Mater..

[2] Li CY, Chai GZ, Yang CC, Wang WF, Xue DS (2015). Tunable zero-field ferromagnetic resonance frequency from S to X band in oblique deposited CoFeB thin films. Sci. Rep..

[3] Kittel C (1947). Interpretation of anomalous larmor frequencies in ferromagnetic resonance experiment. Phys. Rev..

[4] Gau JS, Yetter WE (1987). Structure and properties of obliquedeposited magnetic thin films. J. Appl. Phys..

[5] Lisfi A, Lodder JC, Wormeester H, Poelsema B (2002). Reorientation of magnetic anisotropy in obliquely sputtered metallic thin films. Phys. Rev. B.

[6] Fan XL (2008). *In situ* fabrication of Co_90_Nb_10_ soft magnetic thin films with adjustable resonance frequency from 1.3 to 4.9 GHz. Appl. Phys. Lett..

[7] Yoo JH, Restorff JB, Wun-Fogle M, Flatau AB (2008). The effect of magnetic field annealing on single crystal iron gallium alloy. J. Appl. Phys..

[8] Viala B, Inturi VR, Barnard JA (1997). Effect of magnetic annealing on the behavior of FeTaN films. J. Appl. Phys..

[9] Shokrollahi H, Janghorban K (2007). Different annealing treatments for improvement of magnetic and electrical properties of soft magnetic composites. J. Magn. Magn. Mater..

[10] Yang Y (2010). Influence of the magnetic field annealing on the extrinsic damping of FeCoB soft magnetic films. J. Appl. Phys..

[11] Xi L (2011). Influence of magnetic annealing on high-frequency magnetic properties of FeCoNd films. Mater. Sci. Eng. B.

[12] Nogués J, Schuller IK (1999). Exchange bias. J. Magn. Magn. Mater..

[13] Kuanr BK, Camley RE, Celinski Z (2003). Exchange bias of NiO/NiFe: Linewidth broadening and anomalous spin-wave damping. J. Appl. Phys..

[14] Queste S (2005). Microwave permeability study for antiferromagnet thickness dependence on exchange bias field in NiFe/lrMn layers. J. Magn. Magn. Mater..

[15] Lamy Y, Viala B (2006). NiMn, IrMn, and NiO exchange coupled CoFe multilayers for microwave applications. IEEE Trans. Magn..

[16] Xi L (2010). The high-frequency soft magnetic properties of FeCoSi/MnIr/FeCoSi trilayers. Physica B.

[17] Phuoc NN, Chai GZ, Ong CK (2012). Enhancing exchange bias and tailoring microwave properties of FeCo/MnIr multilayers by oblique deposition. J. Appl. Phys..

[18] Zhan QF, Haesendonck CV, Vandezande S, Temst K (2009). Surface morphology and magnetic anisotropy of Fe/MgO(001) films deposited at oblique incidence. Appl. Phys. Lett..

[19] Ki S, Dho J (2015). Strong uniaxial magnetic anisotropy in triangular wave-like ferromagnetic NiFe thin films. Appl. Phys. Lett..

[20] Briones J (2013). Large area patterned magnetic films by depositing cobalt layers on nano-wrinkled polydimethylsiloxane templates. Appl. Phys. Lett..

[21] Zhang SL (2016). Surface morphology and magnetic property of wrinkled FeGa thin films fabricated on elastic polydimethylsiloxane. Appl. Phys. Lett..

[22] Chai GZ, Phuoc NN, Ong CK (2012). Exchange coupling driven omnidirectional rotatable anisotropy in ferrite doped CoFe thin film. Sci. Rep..

[23] Li SD (2011). Effect of the thickness of Cr interlayer on the high-frequency characteristics of FeCoTa/Cr/FeCoTa multilayers. Adv. Mater. Res..

[24] Phuoc NN, Ong CK (2013). Observation of magnetic anisotropy increment with temperature in composition-graded FeCoZr thin films. Appl. Phys. Lett..

[25] Li SD, Huang ZG, Duh JG, Yamaguchi M (2008). Ultrahigh-frequency ferromagnetic properties of FeCoHf films deposited by gradient sputtering. Appl. Phys. Lett..

[26] Phuoc NN, Ong CK (2014). Gradient-composition sputtering: An approach to fabricate magnetic thin films with magnetic anisotropy increased with temperature. IEEE Trans. Magn..

[27] Phuoc NN, Chapon P, Acher O, Ong CK (2013). Large magneto-elastic anisotropy enhancement with temperature in compositiongraded FeCoTa thin films. J. Appl. Phys..

[28] Phuoc NN, Ong CK (2014). Electric field control of microwave characteristics in composition-graded FeCoTa film grown onto [Pb(Mg_1/3_Nb_2/3_)O_3_]_0.68_-[PbTiO_3_]_0.32_(011) crystal. Appl. Phys. Lett..

[29] Khang DY, Jiang HQ, Huang Y, Rogers JA (2006). A stretchable form of single-crystal silicon for high-performance electronics on rubber substrates. Science.

[30] Chen X, Hutchinson JW (2004). Herringbone buckling patterns of compressed thin films on compliant substrates. J. Appl. Mech..

[31] Huang ZY, Hong W, Suo Z (2005). Nonlinear analyses of wrinkles in a film bonded to a compliant substrate. J. Mech. Phys. Solids.

[32] Jiang HQ (2007). Finite deformation mechanics in buckled thin films on compliant supports. Proc. Natl. Acad. Sci. USA.

[33] Johnson MT, Bloemen PJH, Broeder FJA, Vries JJ (1996). Magnetic anisotropy in metallic multilayers. Rep. Prog. Phys..

[34] Garg SK, Datta DP, Kumar M, Kanjilal D, Som T (2014). 60 keV Ar^+^-ion induced pattern formation on Si surface: Roles of sputter erosion and atomic redistribution. Appl. Surf. Sci..

[35] Chen K, Frӧmter R, Rӧssler S, Mikuszeit N, Oepen HP (2012). Uniaxial magnetic anisotropy of cobalt films deposited on sputtered MgO(001) substrates. Phys. Rev. B.

[36] Stoner EC, Wohlfarth EP (1948). A mechanism of magnetic hysteresis in heterogeneous alloys. Phil. Trans. R. Soc. Lond. A.

[37] Wang ZH, Cristiani G, Habermeier HU (2003). Uniaxial magnetic anisotropy and magnetic switching in La_0.67_Sr_0.33_MnO_3_ thin films grown on vicinal SrTiO_3_(100). Appl. Phys. Lett..

[38] Kondorsky E (1940). On hysteresis in ferromagnetics. J. Phys. (Moscow).

[39] Han XM, Ma JH, Wang Z, Zuo YL, Xi L (2014). In-plane uniaxial anisotropy and magnetization reversal mechanism of FeCo films by strip pattern. Acta Metall. Sci. (Engl. Lett.)..

[40] Suponev NP, Grechishkin RM, Lyakhova MB, Pushkar YE (1996). Angular dependence of coercive field in (Sm,Zr) (Co,Cu,Fe)*z* alloys. J. Magn. Magn. Mater..

[41] Néel L, Pauthenet R, Rimet G, Giron VS (1960). On the laws of magnetization of ferromagnetic single crystals and polycrystals. Application to uniaxial compounds. J. Appl. Phys..

[42] Mathews M, Houwman EP, Boschker H, Rijnders G, Blank DHA (2010). Magnetization reversal mechanism in La_0.67_Sr_0.33_MnO_3_ thin films on NdGaO_3_ substrates. J. Appl. Phys..

[43] Wei JW (2014). An induction method to calculate the complex permeability of soft magnetic films without a reference sample. Rev. Sci. Instrum..

[44] Snoek JL (2014). Dispersion and absorption in magnetic ferrites at frequencies above one Mc/s. Physica.

[45] Acher O, Adenot AL (2000). Bounds on the dynamic properties of magnetic materials. Phys. Rev. B.

[46] Yu Y (2015). Static and high frequency magnetic properties of FeGa thin films deposited on convex flexible substrates. Appl. Phys. Lett..

